# High-level laser therapy versus scalpel surgery in the treatment of oral lichen planus: a randomized control trial

**DOI:** 10.1007/s00784-021-03867-y

**Published:** 2021-03-11

**Authors:** Svetlana Tarasenko, Mikhail Stepanov, Elena Morozova, Alexey Unkovskiy

**Affiliations:** 1 Department of Dental Surgery, Sechenov First Moscow State Medical University, Bolshaya Pirogovskaya Street, 19с1, 119146 Moscow, Russia; 2 Department of Prothodontics, Geriartric Dentistry and Craniomandibular Disorders, Charité-Universitätsmedizin Berlin, Corporate Member of Freie Universität Berlin, Humboldt-Universität zu Berlin, Aßmannshauser Str. 4-6, 14197 Berlin, Germany

**Keywords:** Oral lichen planus, Er:YAG, Nd:YAG, Interleukin-6, Interleukin-1, Interferon

## Abstract

**Objective:**

To compare the clinical effectiveness of various types of high-level laser therapy (HLLT) toward scalpel excision for the surgical treatment of erosive oral lichen planus (OLP).

**Materials and methods:**

The total number of 128 individuals were enrolled in the study. The 35 did not meet the inclusion criteria due to malignancy signs and presence of diabetes mellitus. In total, 8 were lost to follow-up, and 10 were excluded from the analysis, due to analgesics intake. This way 75 patients with the erosive form of OLP were analyzed in three intervention groups (Er:YAG, *n* = 19; Nd:YAG, *n* = 15; Er:YAG + Nd:YAG combination, *n* = 20) and one control group with scalpel excision (*n* = 21). The therapy effectiveness has been assessed based on the comparison of salivary interleukin (IL)-1β, IL-6 and interferon (IFN)-γ preoperative levels to 14, 30 days, and 2 years postoperation, as well as pain level and time of epithelization.

**Results:**

All HLLT groups demonstrated a significantly (*p* > 0.05) higher IL-1β, IL-6, IFNγ and pain level reduction and quicker epithelization toward the control group on the 30th day, except Nd:YAG in case of IFNγ level. The highest IL-1β, IFNγ and pain level reduction and quicker epithelization on the 30th day was observed in Er:YAG group, followed by Er:YAG + Nd:YAG combination, Nd:YAG respectively. However no significant difference was observed between the HLLT groups with regard to IL-6 level reduction. After a 2-year follow-up, no significant difference was observed between all study groups with regard to all variables.

**Conclusion:**

HLLT yields a superior clinical outcome compared to the scalpel excision for the surgical treatment of oral lichen planus, whereby the Er:YAG has been proposed as the most effective laser type at the end of the first postoperative month.

**Clinical relevance:**

For the surgical treatment of erosive OLP the Er:YAG laser may be a preferable treatment option compared to Nd:YAG and scalpel surgery.

**Trial registration:**

The present trial was registered retrospectively in the German Clinical Trials Register, as a member of WHO international clinical trials registry platform, on the 18.03.2020 with the following number: DRKS00020986

## Background

The oral lichen planus (OLP) is a chronic inflammatory potentially malignant mucocutaneous disease, in which immune responses play a major role. The OLP manifests in middle-aged to elderly, mostly female patients, and may be also associated with viral infection, cardiovascular diseases, diabetes mellitus, and thyroid dysfunction [1–3]. The diagnostics of OLP is based on clinical examination and oral biopsy with histopathological verification [4]. For the therapeutic treatment of OLP, the glucocorticoids are commonly used [5]. In order to avoid the systematical and local side-effects of a long-term glucocorticoid use, some other conservative treatment modalities as photodynamic therapy (PDT) and low level laser therapy (LLLT) may be successfully opted [6–8]. However, the treatment efficiency of LLLT for OLP rehabilitation is disputable, and some studies reported a comparable treatment outcome over the use of glucocorticoids [9, 10].

For OLP cases refractory to conservative therapy a surgical approach may be opted, implying an excision of lichenoid lesion [11, 12], which can be afterward covered with a mucosal graft [13]. Alternatively to the scalpel excision, the surgical high-level laser therapy (HLLT) can be opted, which is capable to penetrate the tissue and can be therefore used for ablation. The erbium-substituted yttrium aluminum garnet (Er:YAG) has been successfully applied for excision of OLP [14] and leukoplakia [15]. The other studies demonstrated Er:YAG as a good alternative to knife incision due to a less number of complications and good wound healing [16, 17]. The utilization of neodymium yttrium aluminum garnet (Nd:YAG) for the treatment of precancerous lesions in the oral cavity [18] and frenectomy [19] has been also reported.

Various inflammatory cytokines such as interleukin (IL)-6, IL-1β, and interferon (IFN)-γ have been proven to be associated with the OLP pathogenesis [20–24] and may be used as biomarkers in serum and saliva for monitoring of disease activity and therapeutic response [25]. According to the Tao et al. the whole unstimulated saliva (WUS) may be considered as a valid medium to reflect the condition of the local lesion [26].

To the authors’ knowledge the clinical performance of the HLLT for excision of OLP lesions has not been assessed yet. Thus, the aim of the present study was to compare the efficacy of Er:YAG and Nd:YAG to the traditional scalpel excision in the surgical treatment of OLP. The levels of IL-6, IL-1β, and IFNγ in the WUS, additionally to the level of pain, and time of epithelization, have been chosen as comparison criteria for the clinical outcome evaluation.

## Methods

### Patients recruitment

The patients referred to the department of oral surgery with OLP lesions were examined intraorally. The cytological analysis was performed in order to exclude any signs of malignancy and a blood analysis to detect any sign of hepatitis-B-virus (HBV), human immunodeficiency virus (HIV), herpes simplex virus (HSV), Epstein-Barr-virus (EBV), and cytomegalovirus (CMV). A general examination by an internist was considered to disclose any signs of diabetes mellitus and cardiovascular diseases. Initially all referred patients received a conservative corticosteroid therapy by their internists prior to refer to our department.

The inclusion criteria was the presence of the erosive form of OLP refractory to conservative therapy. This fact was stated after the signs of OLP did not disappear during the last 6 months of corticosteroid medication. The 6-month pause was conducted prior to start any surgical interventions, in order to neglect any possible posteffect of the corticosteroid therapy. The exclusion criteria were any malignance signs, presence of HIV, HBV, HSV, EBV, and CMV, and exacerbation of hypertonia and diabetes mellitus, allergic reactions on the received conservative therapy, and pregnant female patients. The patient selection was performed by the first study operator.

**Fig. 1:**
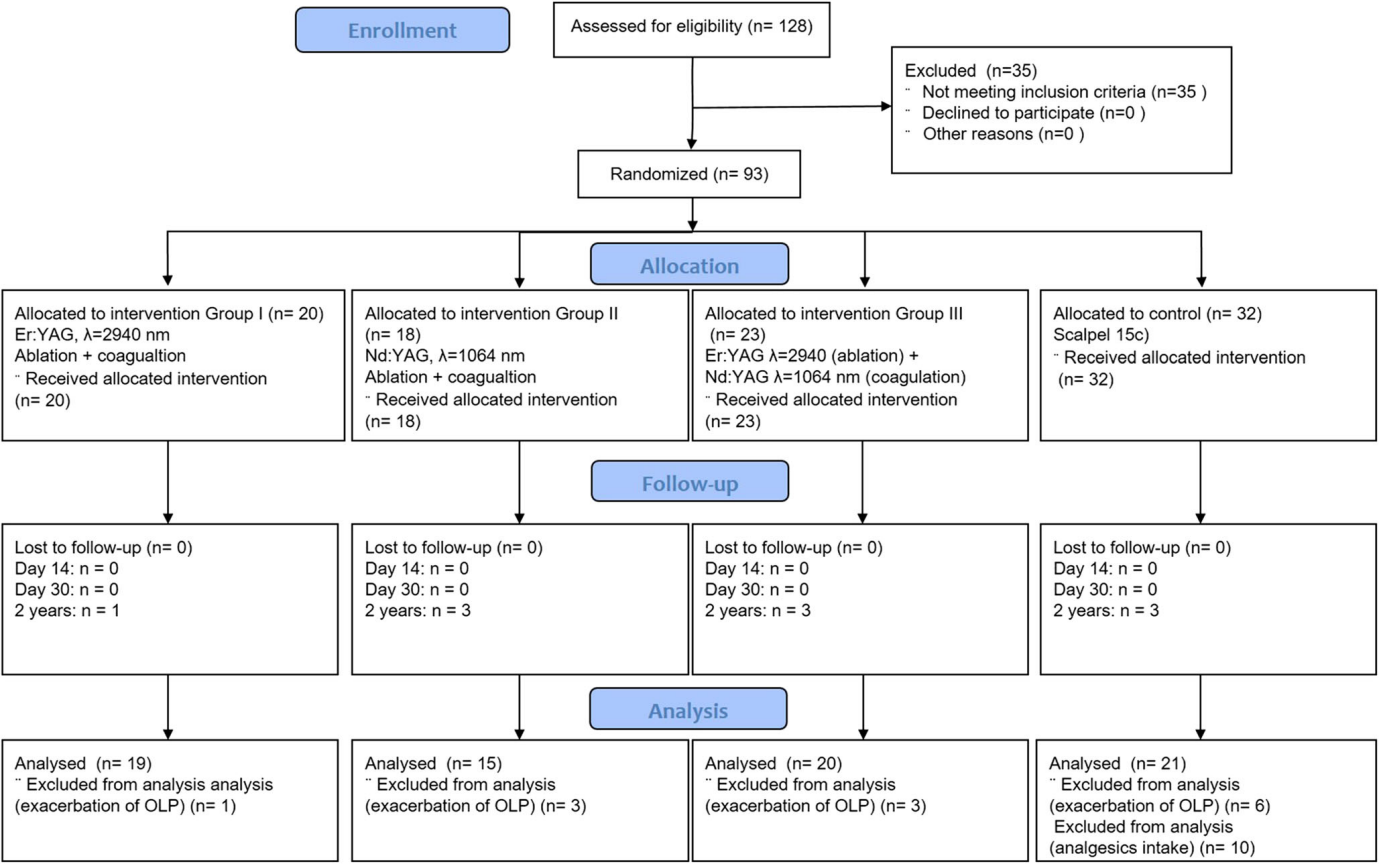
CONSORT flowchart

This way 59 female and 34 male patients with the erosive form of OLP on buccal mucosa, tongue or alveolar ridge have been enrolled in the study and gave their informed written consent for all study measures. The ethical committee of the XXXXXXXXXXX voted affirmatively (xxxxxx). This study was a parallel arm, examiner-masked, randomized control trial (RCT) designed conducted and reported following the Consolidation Standards of Reporting Trials (CONSORT) Statement [27] (Fig. 1).

### Treatment modalities

All 93 male and female patients were randomly categorized into four groups and had the same chances to undergo the surgical treatment either with scalpel incision or with three types of HLLT. Randomization was done by tossing a coin two times in a row, which provided four possible head-tail combinations with accordance to four present study groups. Sealed non-transparent envelopes were used for allocation concealment and opened directly before to the surgical intervention. The surgical treatment was performed by a second study operator.

In the first group the Er:YAG laser (Smart 2940D Plus, DEKA, Calenzano) was used for both ablation and coagulation. The laser parameters are presented in Table 1.

**Table 1:** Laser parameters used in the study

	Group I		Group II		Group III	Group IV
	Er:YAG		Nd:YAG		Er:YAG ablation Nd:YAG coagulation	Scalpel
	Ablation	Coagulation	Ablation	Coagulation		
Wavelength	2940 nm		1064 nm			
Spot size	0.9 mm		300 μm			
Frequency	10 Hz		40 Hz			
Pulse duration	230 μs		350 μs			
Power	2 W	3 W	1.5 W	3 W		
Air–water spray	With	Without	With	Without		
Distance	Contact	1–3 mm		1–3 mm		
Movement types	Reciprocal movements 15 s per cm^2^	Circular movements unfocused beam	Reciprocal movements 15 s per cm^2^	Circular movements unfocused beam		

**Fig. 2:**
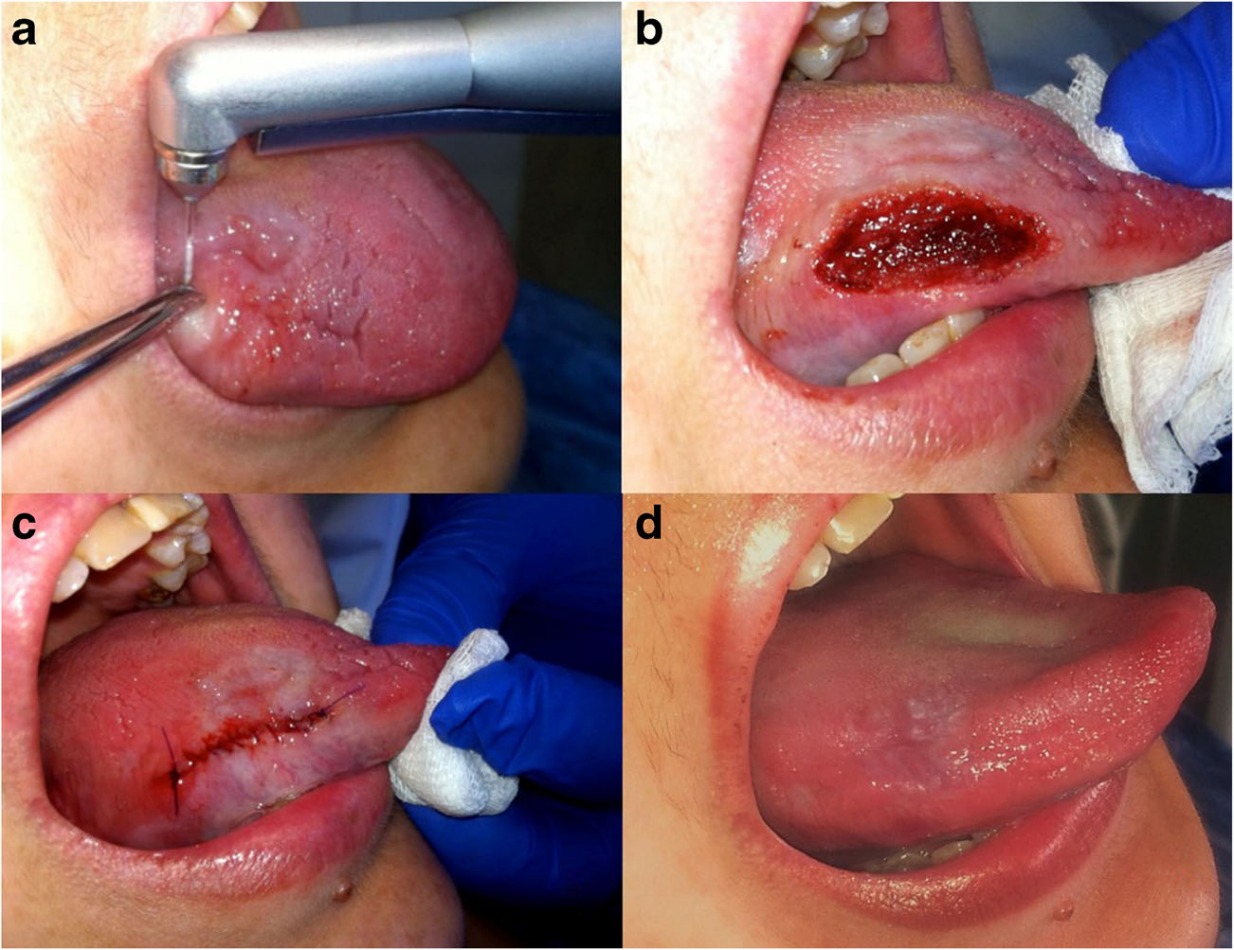
**a** Erosive form of OLP of the tongue being excised with Er:YAG in ablative mode. **b** An extensive wound after being coagulated with Er:YAG in coagulation mode. **c** Stitching of the wound due to the profound defect. **d** The healed wound after a 2-year follow-up without any signs of exacerbation

**Fig. 3:**
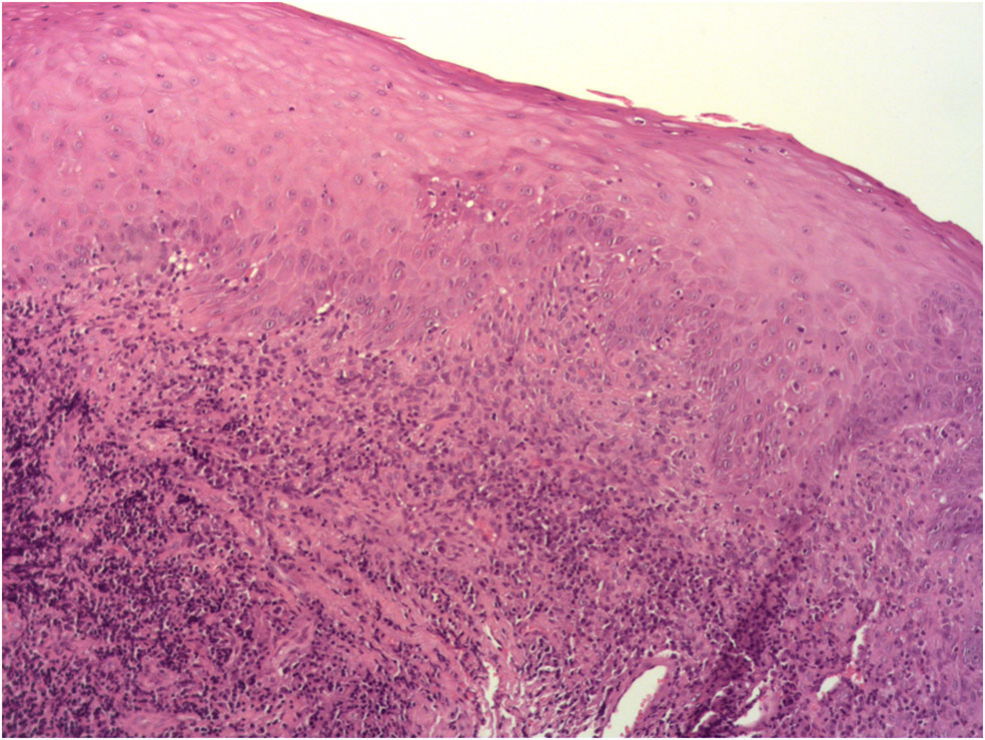
Histopathological verification (hematoxylin and eosin) of the OLP lesion after incisional biopsy (magnification 50×). Presence of plasma cells, subepithelial band-like infiltration of lymphocytes, and acanthosis

In cases of more inward extended erosive lichen planus lesions up to the muscle tissue, the wound was stitched with 5.0 monofilament sutures (Prolene, Ethicon, CA, USA). A clinical example is illustrated in Fig. 2 with histopathological verification in Fig. 3.

**Fig. 4:**
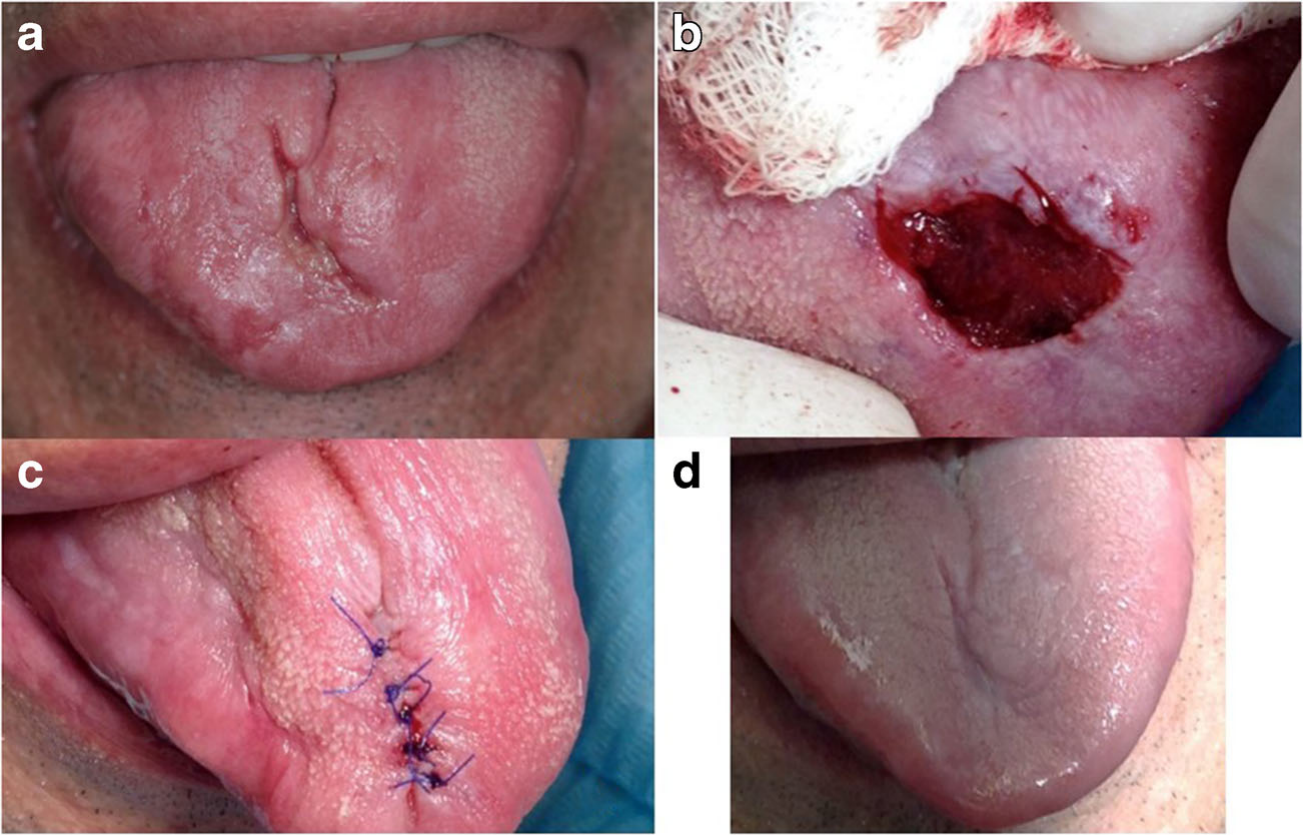
**a** Erosive form of OLP of the tongue. **b** Excision of OLP lesion with Nd:YAG in ablative mode and hemostasis in coagulation mode. **c** Stitching of the wound due to the profound defect. **d** The healed wound after a 2-year follow-up without any signs of exacerbation

**Fig. 5:**
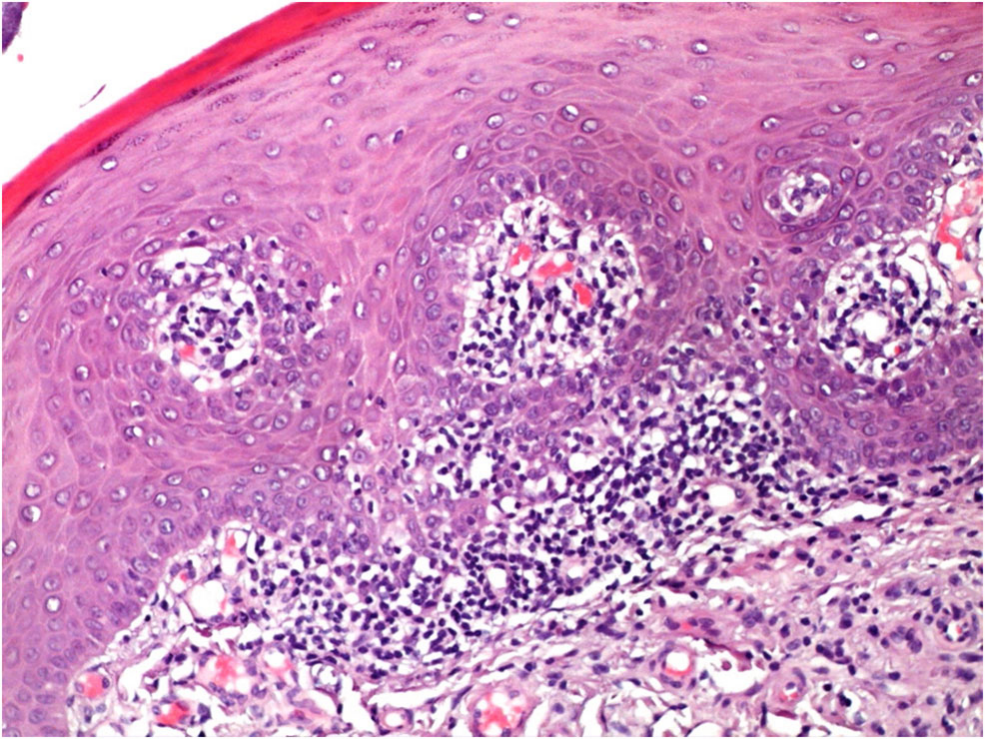
Histopathological verification (hematoxylin and eosin) of the OLP lesion after incisional biopsy (magnification 100×). Presence subepithelial band-like infiltration of lymphocytes

In the second group, the Nd:YAG (Smart File, DEKA, Calenzano) was used for both ablation and coagulation. A clinical example is illustrated in Fig. 4 with histopathological verification in Fig. 5.

In the third group, a combination of Er:YAG in ablative mode and Nd:YAG in coagulation mode (same parameters) was employed.

In the fourth group, the scalpel 15c was performed for the traditional scalpel excision with a subsequent stitching, using approximately 5 to 10 microfilament sutures of 5.0 size (Prolene, Ethicon, CA, USA).

In all patients, the excised tissue portion was taken for the biopsy. Patients were instructed not to ingest any form of analgesic medication during the postsurgical period, except in the case of unbearable pain. In this case, the patients were asked to use ketorolac and analgesic usage was subsequently analyzed.

### Assessment of the treatment efficacy

The treatment efficacy was assessed by comparing the time of epithelization, level of pain, and concentration of inflammatory cytokines IL-1β, IL-6, and IFNγ in the whole unstimulated saliva (WUS) pre-operatively and on the 14th and 30th day postoperatively. Furthermore, the patients were recalled in 2 years for the check of epithelization, signs of fibrosis, and exacerbation. The third operator performed saliva collection, pain, and epithelization assessment, and was unaware, which treatment modality was used by the concrete patient.

#### Collection of oral fluid and cytokine detection

The samples of the WUS were collected by requesting patients to swallow first and then expectorate all saliva into collection tube for 5 min without swallowing. The patients were asked to refrain from eating, using chewing gum, etc. for at least 1 and 1/2 h prior to the appointment. All samples were immediately placed on ice and stored at −20 °C until the analysis was performed.

Indication of inflammatory cytokines IL-1β, IL-6, and Y-IF in the WUS was performed using the enzyme-linked immunosorbent assay (ELISA) [28]. The laboratory crew was unaware, which treatment method was used in each concrete case.

#### Pain

The level of pain was assessed on the 3rd, 14th, and 30th days postoperative using the visual analog scale (VAS). The pain was assessed with the score scale from 0 to 10, where 10 stands for intensively expressed pain, and 0 stands for no pain. The operator was unaware, which treatment method was used in the concrete clinical case.

#### Time of epithelization

Each patient was recalled on the 3rd, 5th, 7th, and 10th days to detect the time of epithelization. Further on, the patients were recalled after 2 years to check for any signs of fibrosis and exacerbation.

#### Lesion extension and distribution

The lesion extension as measured roughly in mm^2^, using the equation for either a circle (*S=πR*^*2*^) or ellipse (*S= πab*), where *π* ≈ 3.14, *R* is the circle radius, *a* is the big ellipse, and *b* is the small ellipse distance. All distances were measured using the sterile ruler. The correlation between the inflammatory cytokines levels and lesion extension was calculated using the Pearson coefficient. The average lesion distribution was calculated for the whole patients’ cohort.

#### Statistical analysis

All gathered data was analyzed with the JMP 13.1 software package (SAS Corp., Heidelberg, Germany). All measurements (cytokines level preoperatively, 14th and 30th days postoperative, 2 years postoperatively; dependent variable) were grouped by the laser type (Er:YAG, Nd:YAG, combination of both, and scalpel; independent variable) and tested for normality by goodness of fit with the Shapiro–Wilk test. As far as the nonnormal distribution within a set of comparisons was revealed, the Wilcoxon rank sum test was used to evaluate statistical difference using alpha = 0.05. The level of significance was set at 0.05.

## Results

128 patients consented to participate in the study. 35 of them did not meet the inclusion criteria. 93 randomized patients underwent the surgical treatment. In all these patients the histopathological verification confirmed the OLP diagnosis (Figs. 3 and 5). No patient was lost during the first and second follow-ups. By the third follow-up, 1 control and 7 intervention subjects were lost due to the OLP exacerbation, and 10 patients were excluded from the analysis, as they have taken the analgesics in the postoperative period after scalpel excision (Fig. 1). The mean lesion extension was 2.23 ± 0.91, 3.25 ± 1.4, 4.17 ± 1.79 and 2.37 ± 0.88 cm^2^ in Er:YAG, Nd:YAG, Er:YAG + Nd:YAG, and control groups, respectively. The lesions were distributed within the oral cavity as follows: 49% on the tongue and buccal plane, 15% on the palate, 15% on the alveolar process, 11% on the mouth floor, and 10% on the lips. The average mean time in ablation mode for each patient was approximately 12 min for Er:YAG and 5.5 min for Nd:YAG.

### IL-1ß

**Fig. 6:**
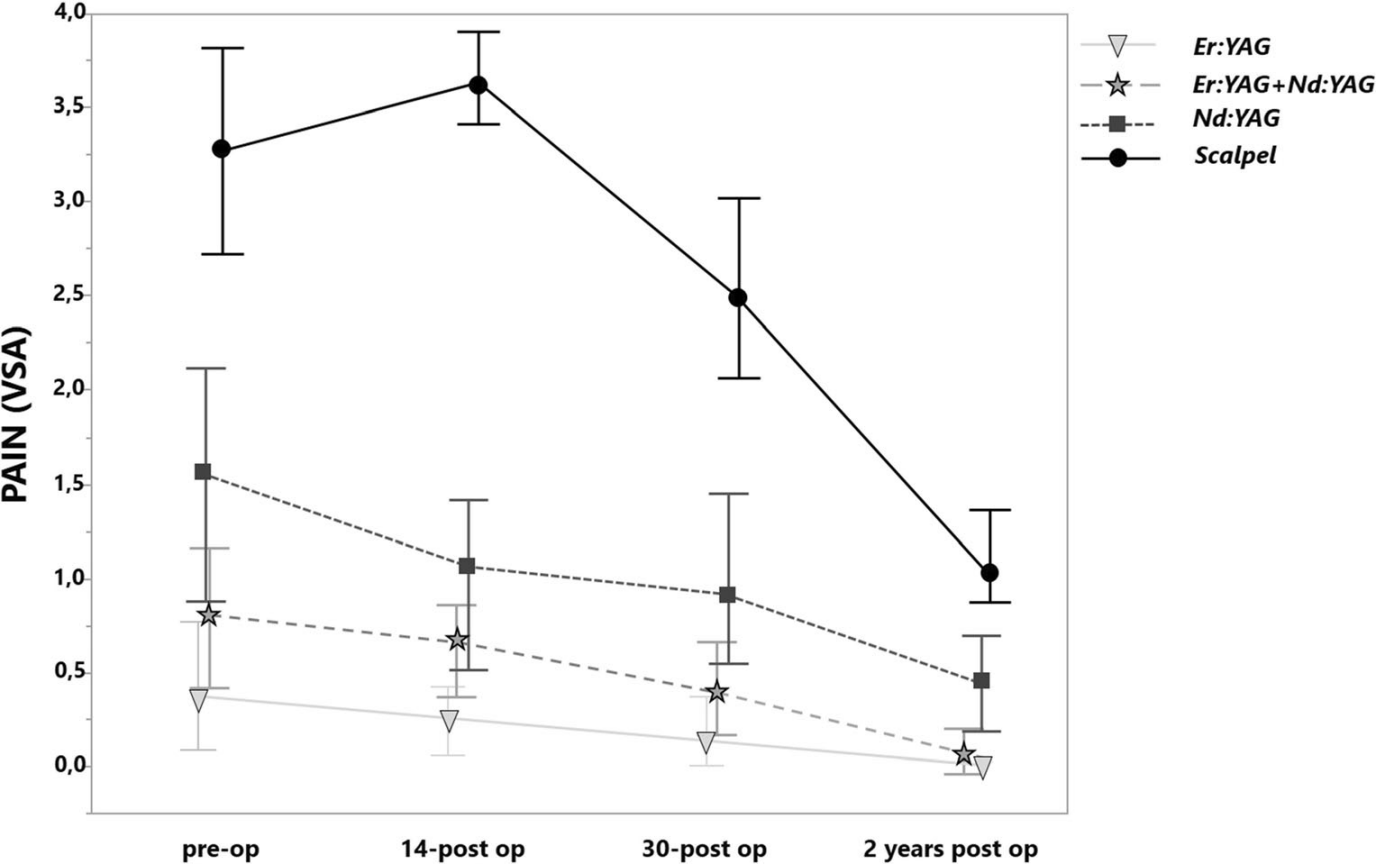
The levels of IL-1β in pg/mL before treatment, on the 14th and 30th days and after a 2-year postoperative treatment in various groups

On the 14th day the level of the IL-1β did not show any significant changes (Fig. 6). On the 30th day in all laser groups, a significant reduction of IL-1β level was observed, whereas in the case of scalpel incision, a slight elevation was detected. The most IL-1β level reduction was observed for the Er:YAG, which differed significantly to Er:YAG + Nd:YAG combination (*p* = 0.0185) and Nd:YAG solely (*p* < .0001). After a 2-year follow-up, no significant difference was observed between all study groups (Table 2).

**Table 2:**
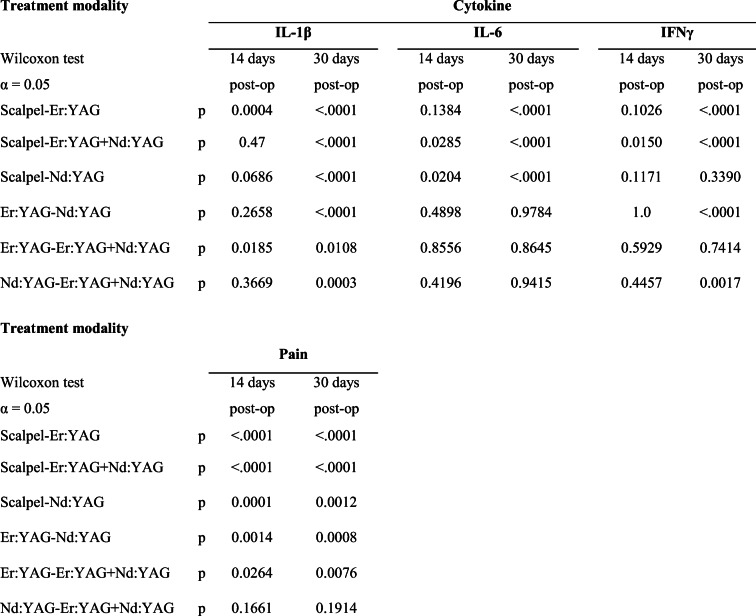
Statistical significance level between each study group (left column) using the Wilcoxon test for cytokine levels (IL-1β, IL-6, and IFNγ) on the 14th and 30th days postop. The 2 years post-operative are not listed as no significant difference was observed between all study groups

The Pearson analysis showed poor positive correlation between the level of IL-1β cytokine pre-operatively and the lesion extension (r = 0.24).

### IL-6

**Fig. 7:**
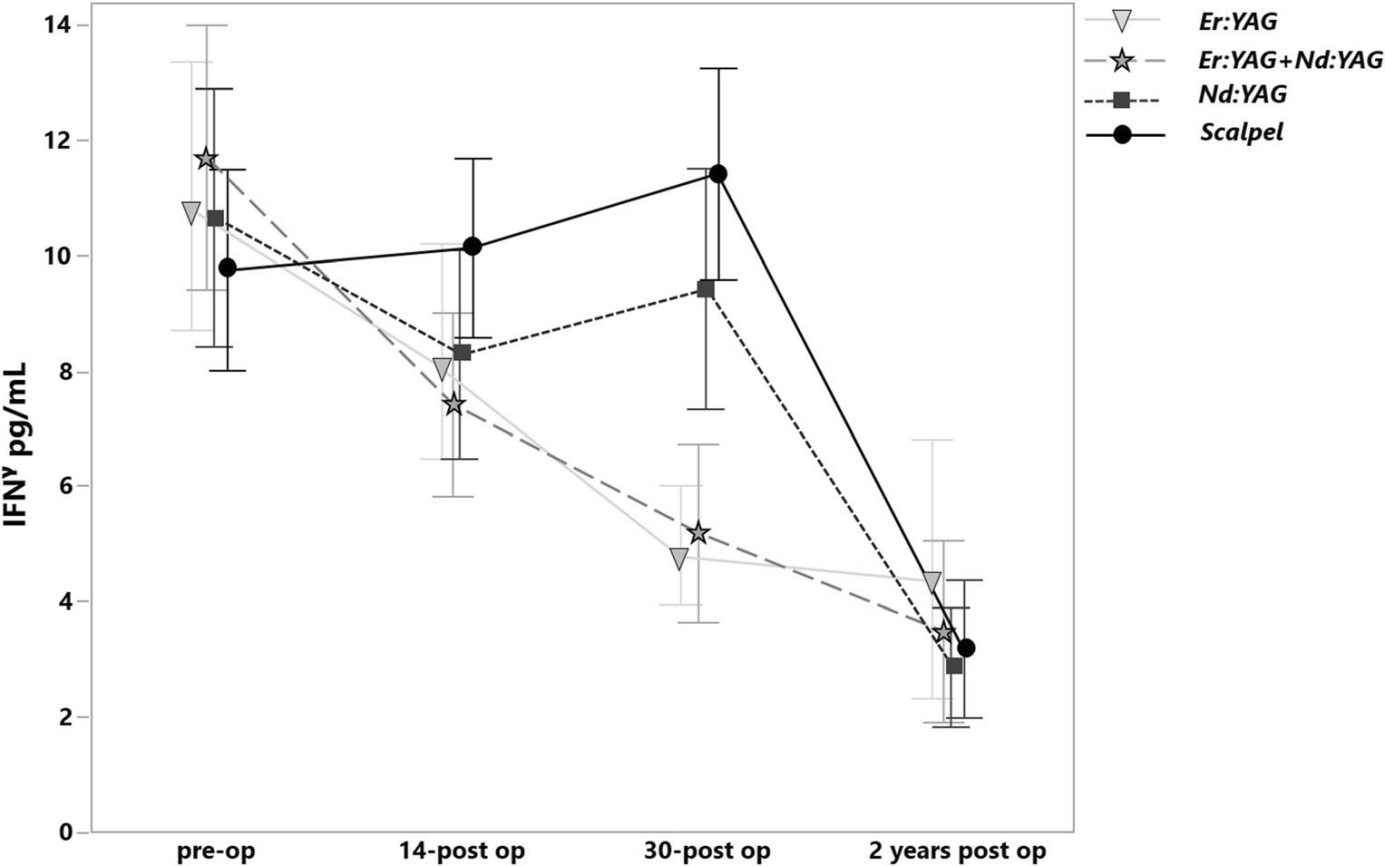
The levels of IL-6 in pg/mL before treatment, on the 14th and 30th days and after a 2-year postoperative treatment in various groups

On the 14^th^ day the level of the IL-6 was slightly decreased for all laser groups (Fig. 7). On the 30th day all laser groups showed significant reduction of IL-6 level compared with scalpel incision (*p* < .0001), whereas again in case of scalpel incision the cytokine level elevation was observed. No significant difference of the IL-6 level was detected between the laser groups (*p* = 0.9). After a 2-year follow-up, no significant difference was observed between all study groups (Table 2).

The Pearson analysis showed no correlation between the amount of the IL-6 cytokine preoperatively and the lesion extension (r = −0.03).

### IFNγ

**Fig. 8:**
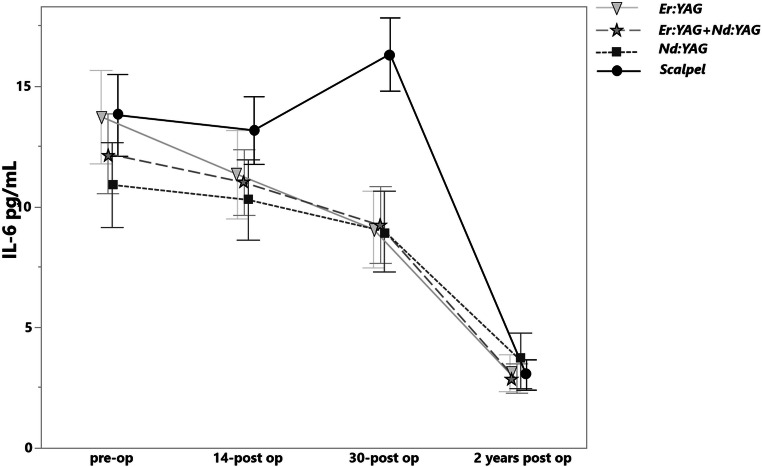
The levels of IFNγ in pg/mL before treatment, on the 14th and 30th days and after a 2-year postoperative treatment in various groups

On the 14th day the level of the IFNγ did not show any significant changes (Fig. 8). On the 30th day the most IFNγ level reduction was observed in the groups of Er:YAG (*p* < .0001) and Er:YAG + Nd:YAG combination (*p* < .0001). In the group of Nd:YAG no significant changes compared with scalpel groups were detected (*p* = 0.3). After a 2-year follow-up, no significant difference was observed between all study groups (Table 2).

The Pearson analysis showed no correlation between the amount of the IF cytokine pre-operatively and the lesion extension (r = 0.07).

### Pain

**Fig. 9:**
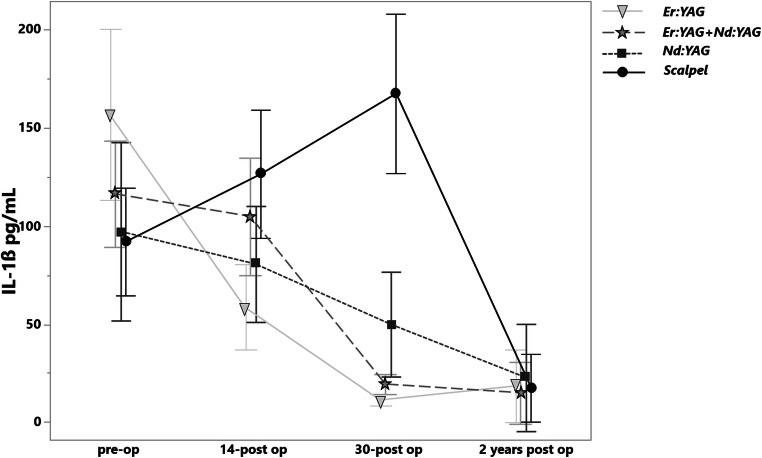
The pain level according to the VAS before treatment, on the 14th and 30th days and fter a 2-year postoperative treatment in various groups

The analysis of pain level showed a significant pain reduction in case of HLLT compared to the scalpel incision (Fig. 9). The most pain level reduction was shown in the Er:YAG group, followed by the combination of Er:YAG + ND:YAG and Nd:YAG groups. After a 2-year follow-up, no significant difference was observed between all study groups (Table 2).

The Pearson analysis showed negative correlation between the pain level pre-operatively and the lesion extension (r = −0.48).

### Epithelization

**Fig. 10:**
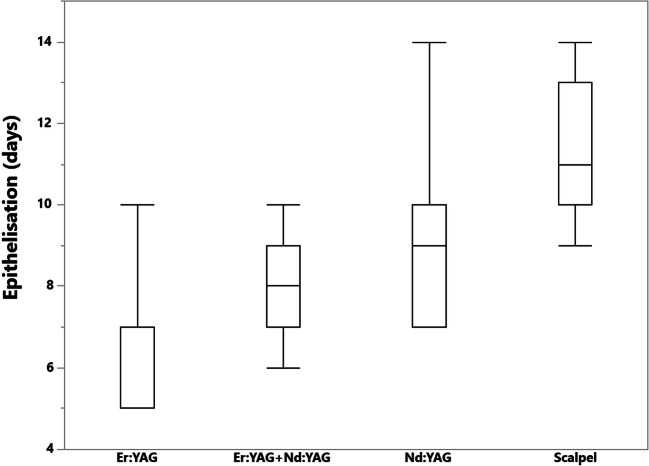
The time of epithelization in days after the Er:YAG, combination of Er:YAG + Nd:YAG, Nd:YAG treatment, and scalpel excision

The epithelization of the wound after the Er:YAG, combination of Er:YAg + Nd:YAG and Nd:YAG treatment was observed on the 6.24, 8.04, 9, and 11.23 days respectively (Fig. 10). The Pearson analysis showed negative correlation between the epithelization time and the lesion extension (r = −0.64).

## Discussion

### Assessment of efficacy based on inflammatory cytokines

Since OLP is an inflammatory disease mediated by T-cells, the detection of inflammation-related cytokines in various medium such as serum and saliva has been the aim of numerous clinical studies. The role of various cytokines in the pathogenesis of the OLP has been described, and they can be used as biomarkers to reveal diagnostic and therapeutic utility in the clinical management of OLP [24].

The association between the IL-6 and OLP has been proven by a row of studies, which reported higher systemic and local IL-6 concentration in patients with OLP compared with healthy groups [22, 29, 30]. The mostly used media were saliva and serum; however, saliva was reported to be more useful for diagnostic and therapeutic aims [23].

IL-1β is an inflammatory cytokine produced mainly by monocytes and macrophages, whose regulatory role in the pathogenesis of OLP has been described in the following review [23]. Some other studies have demonstrated the elevated levels of IL-1β in OLP patients compared to healthy groups and proved the association of IL-1β with the OLP lesion [31–33].

The expression of the IFNγ in the OLP patients has been extensively investigated, and a positive staining of this basic inflammatory cytokine has been observed in the T-cell lines in the OLP biopsies [34]. However, the controversial data was provided on the salivary IFNγ levels. Thus, significantly lower level of IFNγ in patients with OLP was observed in a following study [33]. In contrast, the other studies reported an elevated expression of IFNγ level in the saliva of the OLP patients [26, 34].

### Outcomes of the study

#### HLLT to scalpel

Considering the potential malignant transformation of OLP lesion, the surgical excision has been reported in a few studies [35, 36]. As an alternative to the conventional scalpel excision, the Er:YAG laser was also successfully applied on the patients with OLP. A good and fast healing process and minor discomfort for the patient were reported [14]. The other study has described a positive outcome of the OLP treatment with the Nd:YAG in coagulation mode [7]. The fact that HLLT utilization restricts the histological examination of the lesioned tissue was always considered as its main disadvantage [37]. However, in the present study, the utilization of a thin carbon allowed achieving the coagulation area of 0.05mm. This way the resected lesioned tissues could be successfully sent to the histopathological verification.

Although the utilization of HLLT was reported to deliver a successful treatment outcome, and the scalpel excision for the OLP treatment may be considered as not up-to-date technique anymore, no objective evidence is provided to HLLT utilization in comparison to a traditional scalpel. The findings of the present study demonstrated that both Er:YAG and Nd:YAG utilized in combination of ablative and coagulative mode yielded a superior clinical performance compared to the conventional surgical excision. Thus, in terms of inflammatory cytokines level reduction, the statistically significant difference (*p* < .0001) was observed on the 30th day between all laser groups and scalpel for IL-6 and IL-1β. As for the IFNγ insignificant changes were demonstrated between Nd:YAG and scalpel (*p* = 0.339). In terms of pain level reduction a statistically significant differences (*p* < .0001) were revealed on the 30th day between scalpel and all laser groups. A better clinical performance of HLLT in comparison to traditional scalped excision may be explained by a bloodlessness of operation due the inherent hemostasis effect of the laser, which in its turn enhances the visibility during the operation [17]. A better healing due to less trauma was reported as a common advantage of laser application [38].

With regard to the third follow-up in 2 years, no significant difference was observed between the HLLT and scalpel groups. This means that in long term, both methods may yield a successful treatment outcome.

#### HLLT to each other

The present study revealed significant differences in the IL-1β levels on the 30th day between the laser groups (Table 2). Thus, the Er:YAG showed a superior clinical outcome followed by the combination of Er:YAG + Nd:YAG (*p* = 0.01) and Nd:YAG solely (*p* <.0001). These finding may be attributed to the fact that the Er:YAG has a very shallow thermal penetration of the soft tissue and basically no carbonization occurs, which may explain a good wound healing [28]. This, in its turn, reduces the amount of cytokine expression. Whereas, the Nd:YAG has a deeper effect on the tissue and causes significant lateral tissue damage and subsequent inflammatory reaction, when using it in ablation mode [39]

These cytokine expression level did not correlate with the laser application time, as the average mean time in ablation mode for each patient was approximately 12 min for Er:YAG and 5.5 min for Nd:YAG.

The IL-1β preoperatively levels, observed in the present study were up to 162.58 ± 88.82 pg/mL and coincide with those reported by Jeong et al. by most of the enrolled patients (154 ± 68.9 pg/mL) [40]. The clinical performance of Er:YAG and Nd:YAG for OLP excision has not been compared yet. The present study reported no statistically significant difference on the 30^th^ day in terms of IL-6 level reduction (*p* > 0.09). The Er:YAG, Nd:YAG and the combination of Er:YAG and Nd:YAG on the 30^th^ day showed the salivary IL-6 values of 9.06 ± 3.5, 8.98 ± 3.46 and 9.24 ± 3.77 pg/mL respectively. The data provided on the salivary IL-6 level by healthy groups according to the meta-analysis studies is controversy and is ranging from the 47.46 ± 18.74 to 1.35 ± 1.33 as per Mozaffari et al. [23] and from 110.24 ± 23.78 to 1.05 ± 0.25 pg/mL as per Yin et al. [22]. From this point of view, the patients enrolled in the present study could be considered as recurred. However, the initial IL-6 levels in OLP patients reported in these articles vary dramatically from the present study, so no objective comparison can be performed.

In terms of INFy level reduction on the 30th day the Er:YAG and the combination of Er:YAG and Nd:YAG showed the superior clinical performance than the Nd:YAG (*p* < .0001). The meta-analysis of Mozaffari et al. revealed the heterogeneous salivary IFNγ levels in OLP patients from 0.83 ± 0.54 to 416.67 ± 702.11 pg/mL and in healthy control group from 1.81 ± 0.98 to 52.7 ± 100.51. This data doesn’t coincide with the outcome of the present study and an objective comparison is unfeasible [41]. In contrast, the levels of INFy pre-operatively (from 10.85 ± 4.95 to 12.09 ± 4.81 pg/mL) and 30 days post-operatively (from 5.17 ± 1.99 to 9.96 ± 3.77 pg/mL) are relatively the same as the OLP group (23.95 ± 5.33 pg/mL) and healthy control group from the study of Ghallab et al. (6.41 ± 2.53 pg/mL) [34]. This may indicate the positive relation between the IFNγ salivary level and the treatment response.

In terms of pain reduction significant differences to the Er:YAG were revealed on the 30th day for Nd:YAG (*p* = 0.0008) and Er:YAG and Nd:YAG combination (*p* = 0.0076). A certain positive correlation was observed between the pain and cytokines level reduction as follows: IL-1β (r = 0.59); IL-6 (r = 0.41); IFNγ (r = 0.45). In contrast, a negative correlation (r = −0.48) was observed between the pain level and lesion extension. This fact may be attributed to the lesion depth, which was not taken into consideration, when assessing the lesion extension.

The main hypothesis of using of Er:YAG in ablative mode and Nd:YAG in coagulation mode was related to the fact that Er:YAG utilizes water as chromophore produces a scanty thermal elevation, as only 20% of power is utilized for ablation and the other 80% produce a physiotherapeutic effect [42, 43]. This allows performing the ablation in a more conserve way, than using the Nn:YAG. Whereas the Nd:YAG has a greater affinity to hemoglobin and melanin [39]. This fast is ambivalent. Thus, on one hand, it penetrates the soft tissue in a more aggressive way in ablation mode. On the other hand, in the case of using the Nd:YAG in coagulation mode, the antiinflammatory effect may be guided through the tissue deeper into the lesion, which may also yield a better hemostasis and wound healing. Thus, the main hypothesis of the study was that a more conservative ablation with Er:YAG and more penetrative coagulation with Nd:YAG may comprise an optimal combination promoting an accelerated wound healing. However, the present study demonstrated that such combination of two laser types did not yield any superior clinical outcome.

The 2-year follow-up revealed the normal values of inflammatory cytokines, excepting a few erosive subjects. By 8 subjects, the manifestation of OLP lesion with different localization or either manifestation of diabetes mellitus was revealed.

The present clinical study dealt only with the erosive form of OLP. Some studies have proven than the clinical form of OLP may have an influence of the cytokine expression [26]. The increased levels of IL-1, IL-6 and IFNγ are more associated with the erosive form than reticular and atrophic [29]. Controversially, the study of Dan et al. reported no association between the clinical form and cytokine expression [44].

The OLP lesion localization was not taken into account, when analyzing the cytokine expression and pain level. So no correlation could be encountered between these parameters. This fact should be considered as a limitation of this study, as healing of keratinized mucosa differs from the non-keratinized one.

In the present clinical study the gender distribution was not the same level in all data groups during the recruitment. However, due to the exacerbation of OLP and intake of analgesics 16 more patients were excluded from the analysis, which equalized the patients’ distribution in the study groups.

None of the patients, who was enrolled in the present study and subjected to surgical interventions had any amalgam or gold restorations. These materials are known to case lichenoid reactions if having contact to the oral mucosa [45]. In such cases, eliminating the etiological agent and changing the restoration material would be preferable to any surgical intervention.

## Conclusion

The finding of the present study indicates that the utilization of HLLT is beneficial to the traditional scalpel incision for the surgical treatment of the erosive form of OLP and proposes the Er:YAG to be the most effective HLLT at the end of the first postoperative month among those used in this study. The combination of Er:YAG in ablative mode and Nd:YAG in coagulation mode did not yield any superior treatment outcome.

Within the limitations of the present study, it may be proposed that the levels of IL-1β, IL-6, and IFNγ in the WUS indicate the severity of OLP lesion and may be used in the future as assessment criteria for further treatment modalities.

## Declarations

All patients gave their written informed consent for participation in this study and publication of the gathered data. The study was conducted in accordance with the Declaration of Helsinki.

## References

[1] Di Stasio D, Guida A, Salerno C (2014). Oral lichen planus: a narrative review. Front Biosci (Elite Ed).

[2] Alrashdan MS, Cirillo N, McCullough M (2016). Oral lichen planus: a literature review and update. Arch Dermatol Res.

[3] Baykal L, Arica DA, Yayli S (2015). Prevalence of metabolic syndrome in patients with mucosal lichen planus: a case–control study. Am J Clin Dermatol.

[4] Gupta S, Jawanda MK (2015). Oral lichen planus: an update on etiology, pathogenesis, clinical presentation, diagnosis and management. Indian J Dermatol.

[5] Yang H, Wu Y, Ma H, Jiang L, Zeng X, Dan H, Zhou Y, Chen Q (2016). Possible alternative therapies for oral lichen planus cases refractory to steroid therapies. Oral Surg Oral Med Oral Pathol Oral Radiol.

[6] Mirza S, Rehman N, Alrahlah A, Alamri W’R, Vohra F (2018). Efficacy of photodynamic therapy or low level laser therapy against steroid therapy in the treatment of erosive-atrophic oral lichen planus. Photodiagn Photodyn Ther.

[7] Khater MM, Khattab FM (2019) Efficacy of 1064 Q switched Nd:YAG laser in the treatment of oral lichen planus*.* J Dermatol Treat 1-5 10.1080/09546634.2019.1638881 31328595

[8] Derikvand N, Ghasemi SS, Moharami M, Shafiei E, Chiniforush N (2017). Management of oral lichen planus by 980 nm diode laser. J Lasers Med Sci.

[9] Akram Z, Abduljabbar T, Vohra F, Javed F (2018). Efficacy of low-level laser therapy compared to steroid therapy in the treatment of oral lichen planus: a systematic review. J Oral Pathol Med.

[10] Jajarm HH, Falaki F, Mahdavi O (2011). A comparative pilot study of low intensity laser versus topical corticosteroids in the treatment of erosive-atrophic oral lichen planus. Photomed Laser Surg.

[11] Hadiuzzaman M, Hasibur Rahman M, Parvin Ansari N (2013). A case of recalcitrant oral lichen planus. J Dentis Oral Hyg.

[12] Samal DK, Behera G, Gupta V, Majumdar K, Khurana U (2015). Isolated lichen planus of lower lip: a case report. Indian J Otolaryngol Head Neck Surg.

[13] Axell T, Henriksen BM (2007). Treatment of gingival lichen with free palatal grafts. J Oral Pathol Med.

[14] Fornaini C, Raybaud H, Augros C, Rocca JP (2012). New clinical approach for use of Er:YAG laser in the surgical treatment of oral lichen planus: a report of two cases. Photomed Laser Surg.

[15] Arduino PG, Cafaro A, Cabras M, Gambino A, Broccoletti R (2018). Treatment outcome of oral leukoplakia with Er:YAG laser: a 5-year follow-up prospective comparative study. Photomed Laser Surg.

[16] Broccoletti R, Cafaro A, Gambino A, Romagnoli E, Arduino PG (2015). Er:YAG laser versus cold knife excision in the treatment of nondysplastic oral lesions: a randomized comparative study for the postoperative period. Photomed Laser Surg.

[17] Kumar G, Rehman F, Chaturvedy V (2017). Soft Tissue Applications of Er,Cr:YSGG laser in pediatric dentistry. Int J Clin Pediatr Dent.

[18] Gendelman H, Actis AB, Ouri HO (1993). Neodymium-YAG and CO2 lasers in treatment of pre-cancerous lesions of the oral cavity. Acta Stomatol Belg.

[19] Medeiros Junior R, Gueiros LA, Silva IH (2015). Labial frenectomy with Nd:YAG laser and conventional surgery: a comparative study. Lasers Med Sci.

[20] Parashar P (2011). Oral lichen planus. Otolaryngol Clin N Am.

[21] Nibali L, Fedele S, D'Aiuto F, Donos N (2012). Interleukin-6 in oral diseases: a review. Oral Dis.

[22] Yin M, Li G, Song H, Lin S (2017). Identifying the association between interleukin-6 and lichen planus: a meta-analysis. Biomed Rep.

[23] Mozaffari HR, Sharifi R, Sadeghi M (2018). Interleukin-6 levels in the serum and saliva of patients with oral lichen planus compared with healthy controls: a meta-analysis study. Cent Eur J Immunol.

[24] Lu R, Zhang J, Sun W, du G, Zhou G (2015). Inflammation-related cytokines in oral lichen planus: an overview. J Oral Pathol Med.

[25] Rhodus NL, Cheng B, Myers S, Bowles W, Ho V, Ondrey F (2005). A comparison of the pro-inflammatory, NF-kappaB-dependent cytokines: TNF-alpha, IL-1-alpha, IL-6, and IL-8 in different oral fluids from oral lichen planus patients. Clin Immunol.

[26] Tao XA, Li CY, Rhodus NL, Xia J, Yang XP, Cheng B (2008). Simultaneous detection of IFN-gamma and IL-4 in lesional tissues and whole unstimulated saliva from patients with oral lichen planus. J Oral Pathol Med.

[27] Schulz KF, Chalmers I, Hayes RJ, Altman DG (1995). Empirical evidence of bias. Dimensions of methodological quality associated with estimates of treatment effects in controlled trials. Jama..

[28] Lequin RM (2005). Enzyme immunoassay (EIA)/enzyme-linked immunosorbent assay (ELISA). Clin Chem.

[29] Abdel-Haq A, Kusnierz-Cabala B, Darczuk D, Sobuta E, Dumnicka P, Wojas-Pelc A, Chomyszyn-Gajewska M (2014). Interleukin-6 and neopterin levels in the serum and saliva of patients with Lichen planus and oral Lichen planus. J Oral Pathol Med.

[30] Liu J, Shi Q, Yang S (2017). The relationship between levels of salivary and serum interleukin-6 and oral lichen planus: a systematic review and meta-analysis. J Am Dent Assoc.

[31] Ge Y, Xu Y, Sun W, Man Z, Zhu L, Xia X, Zhao L, Zhao Y, Wang X (2012). The molecular mechanisms of the effect of dexamethasone and cyclosporin A on TLR4 /NF-kappaB signaling pathway activation in oral lichen planus. Gene..

[32] Yamamoto T, Osaki T (1995). Characteristic cytokines generated by keratinocytes and mononuclear infiltrates in oral lichen planus. J Invest Dermatol.

[33] Yamamoto T, Osaki T, Yoneda K, Ueta E (1994). Cytokine production by keratinocytes and mononuclear infiltrates in oral lichen planus. J Oral Pathol Med.

[34] Ghallab NA, el-Wakeel N, Shaker OG (2010). Levels of salivary IFN-gamma, TNF-alfa, and TNF receptor-2 as prognostic markers in (erosive) oral lichen planus. Mediat Inflamm.

[35] Zegarelli DJ (1993). The treatment of oral lichen planus. Ann Dent.

[36] Tamizi M, Moayedi M (1992). Treatment of gingival lichen planus with a free gingival graft: a case report. Quintessence Int.

[37] Vescovi P, Corcione L, Meleti M, Merigo E, Fornaini C, Manfredi M, Bonanini M, Govoni P, Rocca JP, Nammour S (2010). Nd:YAG laser versus traditional scalpel. A preliminary histological analysis of specimens from the human oral mucosa. Lasers Med Sci.

[38] Convissar R (2010) Principles and practice of laser dentistry. Mosby

[39] Romeo U, Russo C, Palaia G (2014). Biopsy of different oral soft tissues lesions by KTP and diode laser: histological evaluation. ScientificWorldJournal..

[40] Jeong SH, Na HS, Park SH, Ahn YW, Chung J (2016). Topical sulfasalazine for unresponsive oral lichen planus. Quintessence Int.

[41] Mozaffari HR, Molavi M, Lopez-Jornet P et al (2019) Salivary and serum interferon-gamma/interleukin-4 ratio in oral lichen planus patients: a systematic review and meta-analysis. Medicina (Kaunas) 55(6) 10.3390/medicina55060257 PMC6630336 31181785

[42] Tamarit-Borrás M, Delgado-Molina E, Berini-Aytés L, Gay-Escoda C (2005). Removal of hyperplastic lesions of the oral cavity. A retrospective study of 128 cases. Med Oral Patol Oral Cir Bucal.

[43] Lubart R, Kesler G, Lavie R, Friedmann H (2005). Er:YAG laser promotes gingival wound repair by photo-dissociating water molecules. Photomed Laser Surg.

[44] Dan H, Liu W, Wang J, Wang Z, Wu R, Chen Q, Zeng X, Zhou Y (2011). Elevated IL-10 concentrations in serum and saliva from patients with oral lichen planus. Quintessence Int.

[45] Thornhill MH, Pemberton MN, Simmons RK, Theaker ED (2003). Amalgam-contact hypersensitivity lesions and oral lichen planus. Oral Surg Oral Med Oral Pathol Oral Radiol Endod.

